# Role of Ayurvedic Principles in Addressing Malnutrition and Non-Communicable Diseases in Low-Resource Settings

**DOI:** 10.7759/cureus.105816

**Published:** 2026-03-25

**Authors:** Manjula, Chhavi Shripat, Maitri Himanshu Nagar, Parameswaran Namboothiri, Jeevan Majgaonkar, Preeti Singh

**Affiliations:** 1 Samhita-Siddhanta (Basic Principles of Ayurveda), BLDEA's AVS Ayurveda Mahavidyalaya, Vijayapura, IND; 2 Department of Community Medicine, Rajasthan University of Health Sciences College of Medical Sciences, Jaipur, IND; 3 Department of Community Medicine, Dr N.D. Desai Faculty of Medical Science and Research, Dharmsinh Desai University, Nadiad, IND; 4 School of Ayurveda, Amrita Vishwa Vidyapeetham, Amritapuri, IND; 5 Panchakarma (Ayurvedic Detoxification and Rejuvenation Therapy), Maharashtra University of Health Sciences, Nashik, IND; 6 Dinacharya and Ritucharya (Lifestyle Interventions), National Post Graduate College, Lucknow, IND

**Keywords:** ayurveda, global health, malnutrition, non-communicable diseases, preventive healthcare

## Abstract

Non-communicable diseases (NCDs) and malnutrition constitute a double burden in low-resource settings, where high out-of-pocket expenditure, inconsistent supply chains, and poor long-term adherence reduce the sustained impact of conventional biomedical interventions. This review critically examines Ayurvedic principles as a complementary, systems-based framework for addressing shared nutritional and metabolic determinants underlying both conditions. Drawing on conceptual foundations and available empirical evidence, the analysis evaluates how dietary regulation, digestive optimisation, lifestyle modification, and *Rasayana*-based preventive strategies can be operationalised using locally accessible foods, herbs, and community-level delivery models. Evidence from South Asian community programs indicates feasibility and relative cost-efficiency, although key limitations remain, including variable study quality, insufficient large-scale trials, and challenges in standardisation and regulation. Rather than positioning Ayurveda as an alternative to biomedicine, the review argues for integrative application aligned with WHO NCD prevention strategies and the Sustainable Development Goals. The review concludes that strategically integrating Ayurvedic preventive principles within existing public health frameworks may strengthen long-term adherence, reduce structural cost barriers, and improve sustainable prevention of malnutrition and NCDs in resource-constrained health systems.

## Introduction and background

Malnutrition and non-communicable diseases (NCDs) are probably the greatest health menace of the 21st century, especially in low-resource settings. Previously, malnutrition was linked to undernourishment, stunting, and susceptibility to infectious illnesses [1]. Low-resource settings denote contexts characterised by limited healthcare infrastructure, constrained financial resources, workforce shortages, and restricted access to specialised nutritional and chronic disease services. However, a major shift is increasingly recognised in global public health: the “double burden of malnutrition,” where undernutrition and micronutrient deficiencies coexist alongside rising overweight and diet-related NCDs within the same populations and communities [2]. In low-resource settings, this overlap creates a compounded risk environment in which nutritional deprivation during the first 1000 days of life (from conception to two years of age) predisposes individuals to impaired growth, altered metabolic programming, and increased risk of adult-onset NCDs, while later-life exposure to obesogenic environments compounds this vulnerability [1]. To a large extent, these two twin epidemics are seen in South Asian, Sub-Saharan African, and Latin American countries, where food insecurity coincides with the diet transition as a consequence of urbanisation [3]. The net result is a health landscape plagued by high morbidity, deaths, and rising expenditures, pushing already shaky health systems to the brink.

There have been some measurable benefits of biomedical interventions, such as micronutrient supplementation and food fortification, drug management, and systematic lifestyle interventions [4]. However, their implementation is hindered in low-resource settings because of a multitude of barriers, including low access to finances, poor healthcare systems, and cultural insensitivity of interventions [2]. Also, the biomedical model is concerned with disease control at an already existing stage, rather than preventive actions involving causal determinants [5]. This creates gaps in health promotion on the long-term and community level. It is thus increasingly being understood that complementary, preventive, and contextually adaptable models must be utilised alongside the traditional models to offer more sustainable solutions [6].

**Figure 1:**
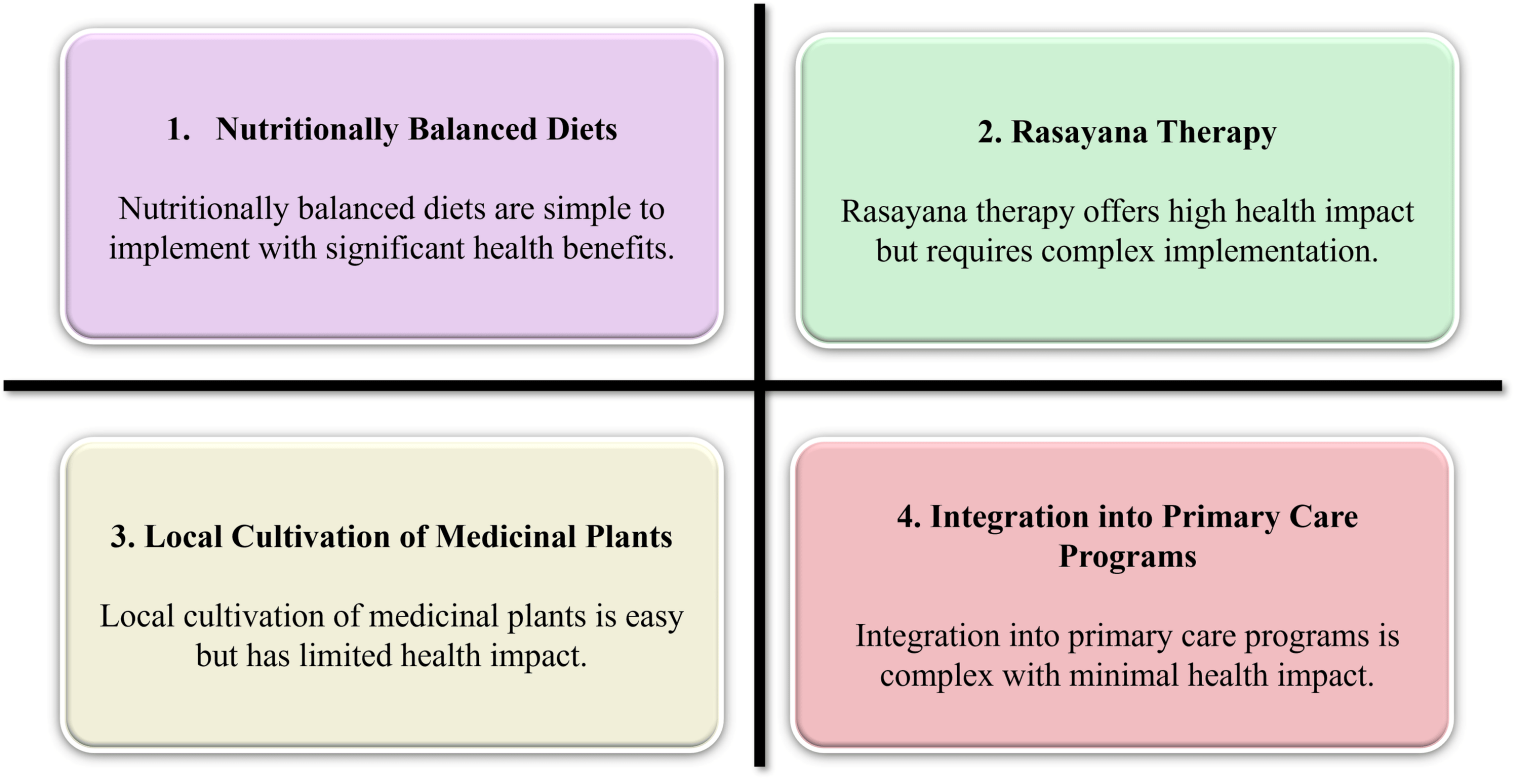
Ayurvedic framework for preventing malnutrition and non-communicable diseases through diet, Rasayana, local plants, and healthcare integration. Image Credit: Authors; created with Microsoft PowerPoint (Microsoft Corporation, Redmond, Washington, United States)

One of the oldest and most complete systems of understanding health in the world, Ayurveda offers a special perspective that can be useful in the current circumstances [7]. Developed in the South Asian peninsula, Ayurveda is now finding its place in the world health literature based on the balance and interdependence of body, mind, and environment [8]. Contrary to models compartmentalising disease, behaviour, and nutrition into distinct categories, Ayurveda places food, living, and environmental conditions at the central point of wellness. Central principles like *ahara* (diet), *agni* (digestive fire), and *ojas* (vital strength) revolve around the role of nutrition and digestion as the core of the body in health promotion and disease prevention [9]. Likewise, the professional focus on *dinacharya* (daily routine) and *ritucharya* (seasonal routine) identifies prophylactic value in customary patterns of living, several of which are low-cost and flexible in cultural contexts [10,11]. Figure 1 presents a conceptual framework showing how Ayurvedic principles translate into practice through dietary strategies, *Rasayana* therapies, locally available plants, and integration into community healthcare systems for the prevention of malnutrition and NCDs.

Surprisingly, these principles can also be applied to modern malnutrition and NCD complications [12]. This holistic and prevention-based orientation aligns directly with the core intent of the United Nations Sustainable Development Goal 3 (SDG 3), Good Health and Well-being, particularly its emphasis on reducing premature mortality from NCDs, strengthening prevention, and improving equitable access to health-promoting interventions [12]. By prioritising low-cost dietary regulation, lifestyle optimisation, and community-based prevention, Ayurveda offers a culturally grounded framework that can support SDG 3 targets in settings where biomedical services alone remain insufficient [13]. This alignment is consistent with the WHO Global Action Plan for the Prevention and Control of NCDs, 2013-2030 [14]. For instance, the food system of Ayurveda, encompassing the six-taste balance (*shad rasa*), provides advice that can reverse the deficiency and excess consumption of nutrients [15]. Ayurvedic nosology presents such disorders as diabetes, hypertension, and obesity as not only clinical disorders but also consequences of lifestyle and environmental imbalance, and the contemporary theories of lifestyle-related chronic disease can be understood [16]. Taking preventive care that is based on Ayurveda, such as yoga, awareness eating, stress management, and exercising, is in line with what the world offers in managing NCDs [17]. In addition, *Rasayana* remedies like Guduchi (*Tinospora cordifolia*), Ashwagandha (*Withania somnifera*), and Amalaki (*Phyllanthus emblica*) improve nutritional hardness and metabolic balance, but more high-quality data is required to confirm their efficacy and safety [18].

Regional adaptability is a key strength of Ayurveda, which often draws on locally available foods and herbs such as Moringa alongside simple lifestyle guidance, making it potentially useful in resource-limited settings [19]. Such interventions may be implemented alongside biomedical programmes, particularly where cost, availability, or cultural constraints limit a conventional approach. Rather than positioning Ayurveda as an alternative to conventional medicine, it can be understood as a complementary framework that integrates evidence-informed traditional practices with biomedical interventions [20]. This integrative strategy may enhance cultural relevance, affordability, and sustainability in addressing the co-burden of malnutrition and NCDs [21].

Ayurveda is a traditional medical system originating in the Indian subcontinent, grounded in a holistic understanding of health as a dynamic equilibrium between physiological, psychological, and environmental factors [22,23]. Its theoretical foundation is structured around the concept of three fundamental biological energies governing the body and mind (*Tridosha*): *Vata* (regulatory and kinetic functions), *Pitta* (metabolic and transformative functions), and *Kapha* (structural and stabilising functions) [24]. These constructs are not anatomical entities but functional descriptors used to interpret patterns of biological regulation. Health is defined as the balanced state of *doshas*, effective digestive and metabolic function (*agni*), adequate tissue nourishment (*dhatu*), proper waste elimination (*mala*), and psychological well-being [9]. Disease is conceptualised as a disturbance of this equilibrium. In the context of malnutrition, impaired digestive-metabolic capacity (*mandagni*) and compromised tissue assimilation are considered central mechanisms, offering a systems-level interpretive perspective that complements biochemical and epidemiological models [25].

The rationale of this review is that it is essential to critically examine how Ayurvedic concepts could be utilised in the population health of low-resource settings. This review does not propose Ayurveda as a replacement for established biomedical or public health interventions for malnutrition and NCDs; instead, it examines Ayurveda as a complementary and preventive approach that may provide culturally contextualised strategies for vulnerable populations [26] The review of Ayurveda as a complementary response to global health problems is highlighted by focusing on the theoretical foundation of its application, empirical evidence, and illustrating how it has been applied in practice [27]. It not only aims to discuss the positive aspects of this system but also to present its constraints, identify gaps in the research, and explore how connections with modern health frameworks may be established [26]. By doing so, it seeks to contribute to the broader discussion on equitable and sustainable health promotion for vulnerable populations at a global scale.

Objectives of the review

This narrative review examines the potential contribution of Ayurvedic principles to the prevention of malnutrition and NCDs in low-resource settings. It outlines the theoretical foundations of Ayurveda relevant to nutrition and chronic disease prevention and critically appraises evidence from clinical trials and community-based interventions. The primary objective is to assess how Ayurvedic dietary, lifestyle, and herbal practices may be integrated into community health systems as supplementary, long-term preventive approaches that complement conventional biomedical interventions.

Methods

This study was conducted as a narrative review aimed at providing an interpretative and critical overview of Ayurvedic approaches relevant to malnutrition and NCD prevention in low-resource settings. The review did not follow a systematic review protocol but adopted a structured literature search to ensure breadth and relevance of sources. A search was performed in PubMed, Scopus, Web of Science, the AYUSH Research Portal, and Google Scholar using combinations of the terms Ayurveda, malnutrition, non-communicable diseases, low-resource settings, dietary interventions, herbal medicine, *Rasayana*, and preventive healthcare, with Boolean operators applied to capture related variations. The search was limited to publications between 2015 and 2025 in order to reflect contemporary research developments and policy discussions.

Eligibility criteria were intentionally broad and aligned with the narrative nature of the review. Publications were included if they discussed Ayurvedic principles or interventions in relation to nutrition, malnutrition, chronic disease prevention, or associated public health outcomes. Eligible sources comprised peer-reviewed journal articles, clinical and observational studies, review articles, and relevant policy documents from recognised organisations such as the WHO and the Ministry of AYUSH. Grey literature and non-English publications were excluded to maintain consistency in quality and interpretative clarity. The selection of studies was based on conceptual and thematic relevance to the objectives of the review rather than on formal quality scoring. No structured risk-of-bias assessment or quantitative pooling was undertaken, as the aim was interpretative synthesis rather than statistical aggregation.

Given the heterogeneity in study designs, intervention types, and reported outcomes, meta-analysis was not performed. Instead, findings were integrated using a thematic narrative synthesis approach, drawing together evidence from clinical studies, community-based interventions, and policy-level discussions. Particular attention was given to feasibility, cultural context, and applicability within low-resource settings.

## Review

Ayurvedic conceptual framework for health and nutrition

Ayurveda is a holistic model of health in which nutrition, physiology, and environment are closely related [16]. It is fundamentally based on the principle of the balance of the three *doshas,* *vata*, *pitta,* and *kapha*, that control both body and mind [24]. Disease is viewed as an expression of a lack of balance, most of the time due to eating errors or environmental pressure. Here, the most important aspect is *agni*, metabolic and digestive energy that transforms food into usable energy and tissues [8]. The lack of *agni* leads to *ama* (toxic waste), a burden on metabolic effectiveness, and a cause of malnutrition and chronic illness [28]. To this is added the concept of *ojas*, the nature of vitality and immunity, which is promoted through proper digestion and food [18]. This model does not express nutrition as a calorie or nutrient source, but as a well-being and strength factor on its own.

Ayurveda, with its emphasis on digestion, assimilation, and systemic balance, provides an interpretive structure that appeals to the present concerns regarding the shortcomings of the calorie approach [5]. In low-resource situations, where the dietary patterns of populations are more likely to be homogeneous and inadequately diversified, this model places high priority on balance, diversity, and cultural acceptability in designing and executing efficient nutrition programs [29]. An important but under-discussed preventive dimension within Ayurveda is *Garbhini Paricharya*, the structured antenatal care regimen described in classical texts [30]. This framework prescribes trimester-specific dietary guidance, behavioural regulation, and lifestyle practices aimed at optimising maternal digestion (*agni*), fetal tissue development (*dhatu* formation), and long-term vitality (*ojas*) [24]. From a contemporary developmental origin of health and disease (DOHaD) perspective, such prenatal optimisation aligns with evidence that maternal nutrition and metabolic status influence fetal programming and subsequent adult-onset risks of diabetes, hypertension, obesity, and cardiovascular disease [31]. Conceptually, *Garbhini Paricharya *therefore represents a life-course preventive model that anticipates modern strategies aimed at reducing NCD risk beginning in utero [30].

**Figure 2:**
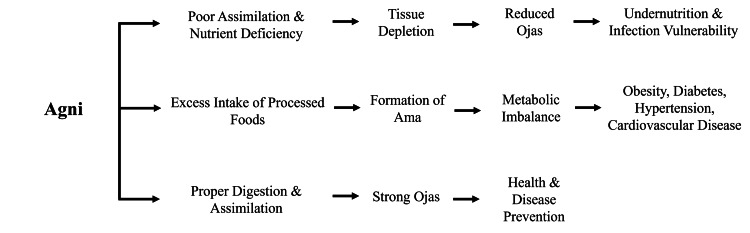
Conceptual model of Ayurveda linking digestive balance with nutritional outcomes Image Credit: Authors; created with Microsoft PowerPoint (Microsoft Corporation, Redmond, Washington, United States) *agni*: digestive fire; *ojas*: vital strength; *ama*: toxic waste

Interestingly, the Ayurvedic system anticipates the co-occurrence of the so-called double burden of malnutrition, i.e., the co-occurrence of under-nutrition and NCDs caused by obesity, which is typical of most of the current low- and middle-income nations [2]. Unlike under-nourishment of protein and energy that depletes tissue stores and weakens immunity, over-consumption of processed foods of poor quality interferes with metabolism, causing diabetes, hypertension, and cardiovascular diseases [3]. Ayurveda prioritises interventions to address the extremes of this continuum by centring agni and metabolic resilience [26]. Figure 2 shows the positioning of *agni *as the mediator between nutritional balance and the continuum from undernutrition to chronic disease.

Dietary principles in Ayurveda and their relevance to malnutrition

Ayurveda recommends dietetic principles (*ahara vidhi*) that are directly applicable to the fight against malnutrition [16]. The most important among them is the theory of the six tastes (shad rasa), namely sweet, sour, salty, bitter, pungent, and astringent, which are purported to have specific physiological effects [15,32]. A well-balanced meal includes all six tastes in their relative proportions and, indirectly, encourages digestion, tissue building, and mental well-being. This is simply restating the same argument from a different perspective, but it is consistent with the prevalent focus in nutrition science on diversity of diet as a central element in avoiding deficiencies in macronutrients and micronutrients [29]. Ayurveda similarly gives great importance to the seasonal and regional variation in diet (ritucharya and deshachar, respectively) [22,33]. Eating food developed according to the local ecosystem and climate is portrayed as tasting better and being better for one's health. This practice, in contemporary global health tradition, reiterates the demands for sustainable diets prioritised for local and indigenous food [34]. With poor-resource groups, the use of locally grown fruits and grains can be cheaper and enhance cultural acceptability, food security, and resilience [21,34].

Protein-energy malnutrition, which is prevalent in most of the rural population, can be explained on Ayurvedic lines as dhata wasting as a result of faulty agni and the absence of proper nourishment [19,35]. It is suggested that the diet be supported by protein-bearing legumes, milk, and clarified butter (ghee) daily with added spice such as ginger or cumin to enhance the process of digestion and absorption [25]. Similarly, the problem of micronutrient deficiencies is constructed as a dietary issue and a metabolic issue. Ayurveda, through its emphasis on digestibility and food combination, clarifies that bioavailability and assimilation, as much as intake, decide nutritional adequacy [8,36]. The principles can be adapted to existing nutrition programs being implemented. Typically, South Asian school feeding schemes incorporating lentils, vegetables, and millets echo Ayurvedic emphasis on digestible and balanced [29]. Comparable interventions may be formulated in African or Latin American environments by augmenting staple-based diets with legumes, greens, and locally available, culturally acceptable spices. Additional Ayurvedic guidance on when to eat, what in moderation, and what to avoid, in combination with biomedical guidance on healthy eating behaviours and taking the right amount, is complementary [26,32].

Ayurvedic approaches to micronutrient deficiencies

Micronutrient deficiencies represent an important barrier to health and development in low-resource settings, and Ayurveda offers several strategies to overcome them through the assistance of diet and medicinal preparations [37]. The most prevalent global nutritional disorder is iron deficiency anaemia, which is addressed by a diet rich in greens, sesame seeds, and jaggery and through the use of medicines such as *Lauha bhasma* (calcified iron) [38]. Preliminary studies have explored *Lauha bhasma *as a potential iron source, although robust clinical validation remains limited. Calcium deficiency, which is a cause of rickets in children and osteoporosis in adults, has been treated for a long time with dietary supplements (dairy products, sesame seeds, leafy vegetables) and mineral preparations (*Shankha bhasma*, a calcined conch shell preparation) [39]. These are often used in combination with stomach stimulants to promote assimilation, reflecting emphasis on the interaction between nutrient intake and metabolism. This general principle has a modern counterpart in the observation that the uptake of calcium is tied to the concentration of vitamin D and to the health of the gastrointestinal tract.

Plants rich in vitamins are also relevant within Ayurvedic nutrition frameworks. Amalaki or Indian gooseberry (*Phyllanthus emblica*), traditionally valued for its rejuvenative properties, is recognised as a natural source of vitamin C and is commonly described in Ayurveda as supportive of immune function and metabolic balance [40]. Similarly, *Moringa oleifera* leaves, widely available in South Asia and Africa, contain substantial levels of vitamin A, iron, and calcium and are generally accessible at low cost. These plants are incorporated into community diets, illustrating the compatibility of Ayurvedic dietary concepts with contemporary nutrition interventions aimed at addressing multiple micronutrient deficiencies simultaneously [41].

A distinctive contribution of Ayurveda is its emphasis that nutritional deficiency may arise not only from inadequate intake but also from impaired digestion and assimilation. Accordingly, Ayurvedic dietary guidance often recommends the use of spices such as ginger, cumin, and black pepper to support digestion and improve nutrient bioavailability [42]. This concept is consistent with modern nutritional evidence showing that vitamin C can enhance iron absorption and that specific food combinations may improve micronutrient uptake. Such convergences suggest that Ayurvedic dietetics may complement biomedical nutrition by emphasising food combinations, preparation, and digestion-related factors, rather than focusing solely on nutrient intake [40].

Nonetheless, limitations must be acknowledged. Most classical formulations are not standardised, and scientific data on their efficacy and safety are variable. While community-based practices of Ayurvedic principles can be a sustainable and culturally acceptable solution, they must be thoroughly tested clinically and carefully regulated to be included in the public health programs [26]. Otherwise, there is a problem of overgeneralization or premature endorsement of interventions that are not necessarily effective universally. Table 1 describes how Ayurvedic treatments for iron, calcium, and vitamin deficiencies match and complement current nutritional practices.

**Table 1:** Ayurvedic approaches to selected micronutrient deficiencies in low-resource settings

Micronutrient Deficiency	Ayurvedic Dietary Sources	Ayurvedic Preparations	Digestive Enhancers (to improve assimilation)	Modern Biomedical Parallel	Reference
Iron (Anemia)	Jaggery, sesame seeds, leafy greens	Lauha bhasma (calcined iron)	Black pepper, ginger, cumin	Vitamin C enhances iron absorption	[38]
Calcium (Rickets/Osteoporosis)	Dairy, sesame seeds, leafy vegetables	Shankha bhasma (conch shell)	Often combined with digestive stimulants	Absorption linked to Vitamin D and gut health	[39]
Vitamin C (Immunity)	Amalaki (Indian gooseberry)	Rasayana preparations	Not specified	Recognised antioxidant and immune booster	[18,40]
Vitamin A, Iron, Calcium	Moringa oleifera leaves	Not specified	Not applicable	Widely used in fortification and supplementation	[41]

Ayurvedic perspectives on NCDs

The prevalent reason for worldwide mortality is NCDs such as diabetes, cardiovascular conditions, hypertension, and obesity, but with disparate prevalence within low- and middle-income nations [43]. Cost, facility, and lifetime drug treatment may be restrictive in these settings. Ayurveda has a unique model of perception of NCDs, one more preventive in nature, regulation of lifestyle, and harmonisation of the system [16]. Diabetes, also known as *madhumeha* in Ayurveda, is a well-known example. This disease is caused by an inefficient metabolism and an excess of heavy and sweet foods. The Ayurvedic model focuses on *kapha dosha* imbalances and poor digestion, which can disrupt the body's energy-regulating system [44]. Insofar as it emphasises the broader interplay of metabolism, behaviour, and diet, this realisation is complementary to the biomedical model of insulin resistance rather than an analogy. Similar to this, obesity is defined as the pathological over-accumulation of adipose tissue (*meda dhatu*) brought on by an unbalanced diet and insufficient exercise [17].

Although hypertension is not mentioned in the ancient texts, it could be explained by an imbalance between *vata* and *pitta*, which is made worse by stress and unhealthy habits. The cardiovascular diseases are idealised as a consequence of the build-up of toxins and poor circulation. The strength of these descriptions lies in that they are preventative [26]. NCDs have been viewed as diet- and exercise-related diseases that can be minimised through controlling diet, physical activity, and restoration of digestive and metabolic equilibrium. Such perception has been in concordance with the recent priorities of public health, seeking factors of disease causation, environmental and behavioural, particularly in low-resource settings where drug-based interventions could be unrealistic [43].

*Rasayana* therapy for prevention and immunity

One of the distinguishing Ayurveda therapies, *Rasayana* therapy, is intended for immune reconstruction, metabolic augmentation, and rejuvenation [45]. Preventive practice is also included in it, which not only gets implemented for malnutrition but also for chronic diseases, putting health promotion within daily living, as compared to clinical practice [42]. Some *Rasayana *plants have been a subject of interest among researchers. Ashwagandha is widely discussed in Ayurvedic literature as a *Rasayana* plant traditionally used for strengthening and vitality [18]. Guduchi is traditionally described as having immunomodulatory properties within Ayurvedic literature. Vitamin C-rich amalaki has been shown to lower cholesterol and oxidative stress, which is consistent with preventing cardiovascular disease [18]. *Chyawanprash* is a traditional preparation with numerous *Rasayana* components that are being utilised to enhance immunity and vitality [45]. Preliminary investigations suggest antioxidant and cardioprotective properties [36]. The focus on community and the preventative approach of *Rasayana *therapy is especially convincing in the conditions of limited resources [20].

In order to reduce the use of imported drugs, certain herbs are to be cultivated locally, consumed in food, and processed by using cheap techniques [46]. Their mutual effect on high immunity and metabolic health can be helpful, especially in an environment where there is a combination of the presence of infectious disease along with an increase in NCDs. However, priorities should be laid on controls. The data of clinical trials is still awaiting, and the problem of small numbers, diversified methodology, and lack of standardisation exists [10]. The main concern is safety problems, dosing problems, and quality control problems. Otherwise, *Rasayana *interventions can be equated in their effectiveness or excessively used, which invalidates them, unless there is robust validation and regulatory control. This is why an individual must consider Rasayana as a complementary approach that is more of an add-on to biomedical therapy, but not as an alternative to it, but as something to be subjected to closer examination [47].

Lifestyle Interventions: *dinacharya* and *ritucharya*

Ayurvedic preventive principles are operationalised as low-cost lifestyle practices through daily (*dinacharya*) and seasonal (*ritucharya*) regimens [10]. These approaches emphasise routine, behavioural regularity, and alignment with environmental cycles, which align with concepts discussed in circadian biology, stress regulation, and lifestyle medicine [48]. *Dinacharya* includes early rising, personal hygiene and cleansing, physical activity, oil massage (abhyanga), regulated meal timing, and adequate rest. Collectively, these practices aim to support mental balance and optimise digestion and metabolism [42]. Several components have biomedical parallels, such as sleep hygiene, structured physical activity, and stress management, which are associated with reduced risk of obesity, diabetes, and cardiovascular disease [43].

**Figure 3:**
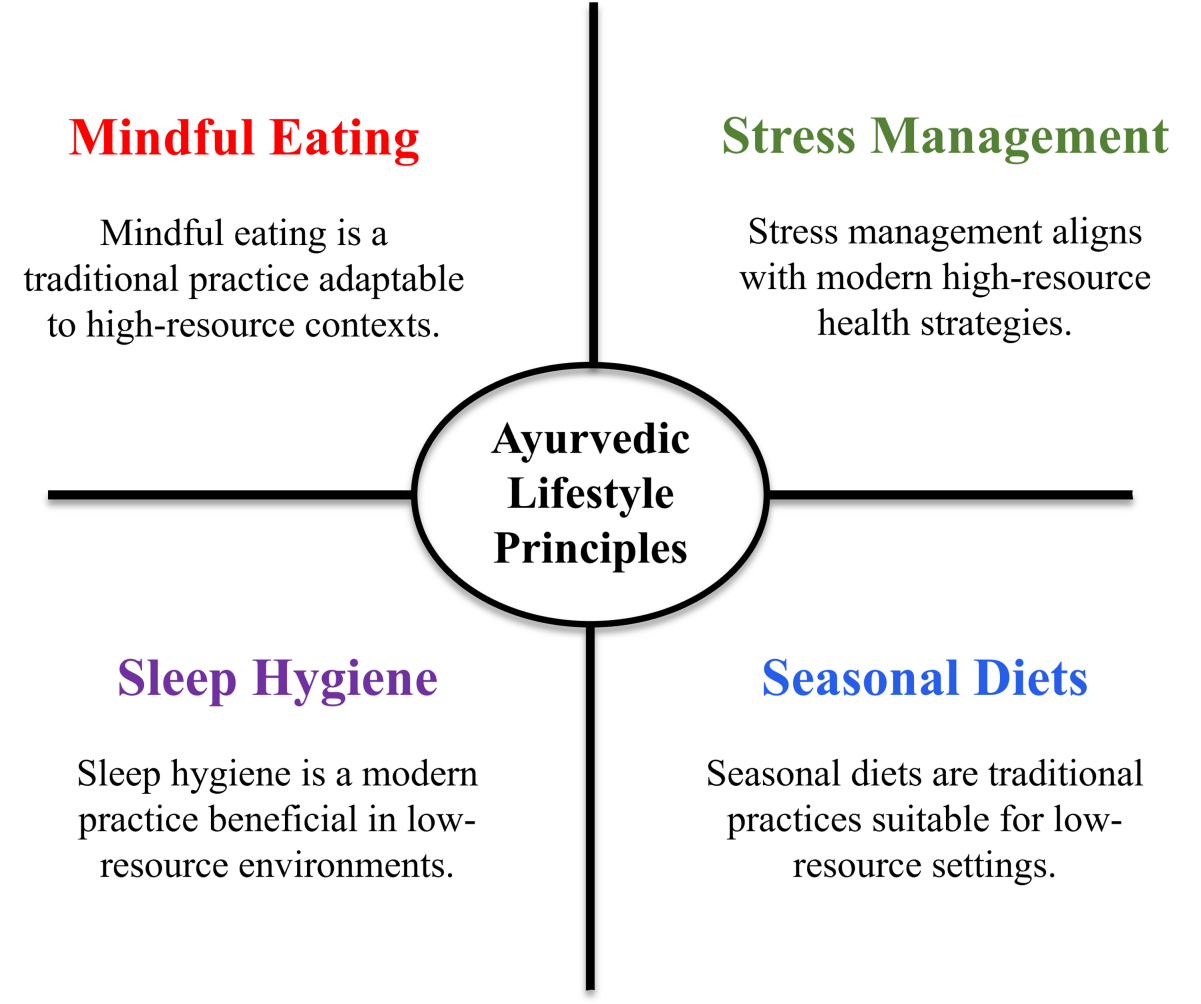
Ayurvedic lifestyle practices and their biomedical parallels Image Credit: Authors; created with Microsoft PowerPoint (Microsoft Corporation, Redmond, Washington, United States)

*Ritucharya* recommends adapting diet and activity to seasonal variations. For example, lighter foods and cooling measures are suggested during hot seasons, whereas heavier and warming foods are recommended in cold seasons [22,33]. This seasonal adjustment can be interpreted in modern terms as encouraging locally available and seasonal foods, which may also support sustainability. In resource-limited settings, ritucharya has been proposed as a practical strategy to optimise nutrition using seasonal availability rather than relying on costly external supplementation. Ayurvedic lifestyle guidance also includes mind-body practices such as yoga and pranayama. *Yoga Nidra* has been reported to reduce blood pressure, improve glycaemic control, and support weight management, while pranayama is used for stress reduction and autonomic balance [49]. Similarly, moderated fasting practices described in Ayurveda can be conceptually compared with intermittent fasting approaches, which have been linked to improved insulin sensitivity and cardiovascular health [50]. Overall, these interventions require minimal infrastructure and can be delivered through community-based education, making them potentially suitable for low-resource populations. As exemplified in Figure 3, Ayurvedic daily and seasonal regimens considerably overlap with contemporary preventive health practices.

Community-based Ayurveda models in low-resource settings

One of the characteristic Ayurveda therapies, *Rasayana* therapy, targets immune development, metabolic augmentation, and rejuvenation [45]. It also offers prevention useful in malnutrition, as well as in chronic diseases, inserting health promotion into the daily life situation, not clinical practice per se. A number of *Rasayana *herbs have caught the attention of scientists. Ashwagandha is a thoroughly documented adaptogenic herb, and the evidence that it is useful for stress management, glucose metabolism, and inflammation has been presented [18]. Guduchi has been studied to be immunomodulatory and antioxidant, and possibly has use in infection resistance and metabolic health [17,18]. Amalaki has high levels of vitamin C, which has been shown to lower oxidative stress and cholesterol, consistent with the prevention of cardiovascular disease [45,50]. *Chyawanprash*, a multi-ingredient traditional preparation with several *Rasayana *ingredients, is still commonly used as an energiser and immunostimulant, and preliminary trials show antioxidant and cardioprotective action [7,45].

The community-oriented and preventive approach of *Rasayana *therapy is also attractive in the low-resource environment [51]. Many of these drugs must be feasible to cultivate locally, added to food, and formulated in low-cost preparations so that dependency on foreign drugs can be reduced [46]. Their overall role in conferring immunity and metabolic wellness is especially relevant in the environment where both infectious diseases and increasing NCDs are present. But restrictions must be placed in a global perspective [32]. Results of clinical trials need to be smoothed out, and there are concerns of small sample sizes, methodological diversity, and lack of standardisation. Safety concerns, dosing, and issues of quality control come into the limelight. *Rasayana *treatments can only be equalled in effectiveness or become depleted to the extent of losing face value without needing evidence and control [10]. All such reasons make one consider *Rasayana* not a replacement for biomedical therapy but an added method of practice that should be subjected to more rigorous research.

Cost-effectiveness and accessibility of Ayurvedic interventions

Ayurvedic interventions are often considered cost-effective in low-resource settings because many core preventive recommendations rely on behavioural modification rather than specialised equipment, pharmaceuticals, or advanced infrastructure [12]. *Dinacharya* and *ritucharya* routines translate preventive theory into structured, low-cost habits that emphasise balance and alignment with natural rhythms, concepts that are increasingly discussed in circadian biology, stress management, and lifestyle medicine research [5]. Apart from the simple daily practices mentioned earlier, several components also correspond to established biomedical prevention strategies such as sleep hygiene, regular exercise, and stress control, which are linked with reduced risk of obesity, diabetes, and coronary artery disease [11]. *Ritucharya* focuses on adjusting diet and activity according to seasonal variation [22]. In resource-poor communities, *ritucharya* has been discussed as a practical framework for optimising nutrition based on seasonal availability and reducing reliance on expensive external supplementation [33]. Ayurveda also incorporates mind-body approaches such as yoga and pranayama. Evidence has indicated that yoga may support blood pressure regulation, glycaemic control, and weight management, while pranayama is used to regulate stress and autonomic balance [8]. Moderated fasting described in Ayurveda has also been conceptually compared with intermittent fasting, which has been associated with improvements in insulin sensitivity and cardiovascular risk factors [27]. Overall, these approaches are accessible because they can be implemented through community-based education with minimal infrastructure, making them potentially suitable for low-resource communities. Table 2 enumerates Ayurvedic daily practices and their biomedical equivalents, highlighting their preventive use in low-resource populations.

**Table 2:** Ayurvedic lifestyle practices and their biomedical parallels in preventive health

Ayurvedic Practice	Key Components	Biomedical/Modern Parallels	Preventive Health Benefits	Reference
Dinacharya (Daily Regimen)	Waking before sunrise, hygiene, physical activity, oil massage (abhyanga), mindful eating, adequate rest	Sleep hygiene, regular exercise, and stress management	Supports metabolic activity, improves digestion, and reduces the risk of obesity, diabetes, and cardiovascular disease	[10,42]
Ritucharya (Seasonal Regimen)	Seasonal diet and activity (cooling foods in summer, warming foods in winter, reliance on local produce)	Sustainable nutrition, seasonally adapted food policies	Maximises nutrition in resource-scarce contexts, reduces dependence on supplementation, and enhances resilience	[10,22]
Yoga	Postures, breathing–movement integration	Physical activity, mind–body exercise	Reduces blood pressure, supports weight management, and improves glycemic control	[9]
Pranayama	Regulated breathing exercises	Stress management, autonomic balance practices	Improves cardiopulmonary function, reduces stress, and enhances mental health	[49]
Moderate Fasting	Periodic, mindful restriction of food intake	Intermittent fasting	Improves insulin sensitivity, supports cardiovascular health, and promotes metabolic balance	[51]

Integrating Ayurveda with modern public health programs

Ayurveda must be integrated into the modern public health programs so that it can bring in the maximum benefit, rather than operating independently [6]. Such integration is concurring with global movements like the WHO universal health coverage and SDGs that recognise cost, access, and cultural appropriateness as main drivers [12]. The WHO Traditional Medicine Strategy 2014-2023 recognises the role of traditional and complementary medicine within UHC frameworks, emphasising integration, regulation, safety, and evidence-based evaluation [52]. This policy framing strengthens the relevance of Ayurveda to public health systems, particularly in low-resource settings [8]. India has been leading such integrations for the first time [46]. AYUSH professionals are integrated at the level of primary health centres with the help of the National Health Mission, thus enhancing service delivery in rural areas [53]. School nutrition programs have tried out Ayurvedic ingredients such as millets, fruits, and herbal dietary supplements, which are add-ons to biomedical dietary recommendations [27]. Biomedical information regarding an equilibrated diet is normally complemented by Ayurvedic advice on mindful eating, yoga, and seasonal modulation, which boosts participation and follow-up of the population [9].

Worldwide, Ayurveda's preventative philosophy is attuned to the needs of whole-of-society strategies for preventing NCDs. Its focus on dietary variety, control of life, and inexpensive treatment modalities is aligned with the SDGs' requirement of sustainable and culturally-sensitive health practice [14]. The collaborations between Ayurvedic schools and international health organisations would lead to comparative information and policy [15]. However, integration means closing epistemological gaps. Ayurveda and biomedicine employ different conceptual languages, and it is essential to develop communication and evaluation systems [30]. The integration of these practises will not be a formality as the referral procedures, evidence-based validation, and definite standards of practise will be developed. In their absence, disintegration is rather a threat than an integration. Table 3 states the integration of Ayurveda into the public health agenda, how it is aligned with the global health agenda, and the benefits and drawbacks of such efforts.

**Table 3:** Integration of Ayurveda with contemporary public health programs AYUSH: Ayurveda, Yoga and Naturopathy, Unani, Siddha, and Homoeopathy; WHO: World Health Organization; UHC: Universal Health Coverage; SDG: Sustainable Development Goals; SDGs: Sustainable Development Goals; NCD: Non-Communicable Diseases

Domain	Current Integration Examples	Global Health Alignment	Benefits	Challenges	Reference
Primary Healthcare	AYUSH practitioners integrated into India’s National Health Mission primary health centres	WHO UHC goals	Expands rural service delivery; improves access	Requires standardised training and referral systems	[53]
School Nutrition Programs	Inclusion of millets, local fruits, and herbal supplements in mid-day meals	SDG focuses on sustainable nutrition	Enhances dietary diversity and cultural acceptability	Needs rigorous evaluation and safety monitoring	[14,27]
Community Engagement	Combining biomedical diet messages with Ayurvedic guidance (mindful eating, yoga, seasonal adaptation)	WHO NCD prevention strategy	Improves adherence and community participation	Risk of inconsistent messaging if not harmonised	[12]
Global Partnerships	Potential collaborations between Ayurvedic institutions and international health agencies	SDGs & whole-of-society approaches	Generates comparative data, fosters policy adoption	Conceptual and epistemological differences remain	[27]

Challenges, evidence gaps, and ethical considerations

Ayurveda is a profitable commodity; however, there are huge obstacles to Ayurveda being introduced to global healthcare [54]. The first problem is the gap in the quality of evidence. Although several small-scale studies have indicated the possible advantages of Ayurvedic herbs and practises, there is still a lack of large randomised controlled studies [10]. This undermines Ayurveda in evidence-based policy making and inclusion in the global guidelines [8]. The second important concern is standardisation [46]. The Ayurvedic preparations vary in their mode of preparation, dosage, and the material used. The fact that some traditional medicines are being contaminated with heavy metals has raised some concerns, and therefore, quality control is necessary and should be monitored by the regulating bodies. In the absence of standardised protocols, traditional Ayurvedic interventions can be scaled up, albeit at the cost of inefficacy and damage [53].

There is also the issue of cultural sensitivities. Ayurveda is easily accepted in South Asia, but outside it can be suspiciously or ignorantly accepted. In countries that already have indigenous health systems, standardised Ayurvedic preparations can be seen as being imposed on them. Successful implementation would therefore require being undertaken with proper respect to the community, giving respect to indigenous knowledge, and with open discussions on the risks and benefits [20]. Finally, there are ethical issues. There is an urgent need not to exaggerate the benefits of Ayurveda or present it as a substitute for the required biomedical care [26]. For instance, while *Rasayana* herbs can be used to preserve metabolic health, they cannot replace insulin therapy in type 2 diabetes [55]. Distortion can delay critical therapies and lead to injuries. Ethical integration involves the placement of Ayurveda as an add-on in a way that the patients can continue to receive biomedical services for acute or severe illness [20].

Limitations and future considerations

Current evidence in support of Ayurveda in the treatment of malnutrition and NCGs is constrained by a few limitations. Numerous current studies are small, local, and lacking in follow-up longitudinal analysis, and thus restrict generalizability to populations with heterogeneity. Deficiencies in methods, including a lack of focus on the utilisation of randomised controlled designs, a skewed choice of outcomes, and a lack of blinding, also reduce reliability. Standardisation and quality control are likewise important concerns: Ayurvedic products can differ in ingredients and quantity, and there are frequent findings of adulteration that can impact safety. Potential ventures are community-based interventions and pilot projects, although the level of evidence is not constant, and it is challenging to declare them universally applicable to the international health community.

The remaining areas of deficit would need to be addressed by subsequent large, properly conducted studies and cross-country comparisons of Ayurvedic treatments across varying cultural and socioeconomic backgrounds. There has to be standardisation of formulae, strictness in quality control, and regulatory control to ensure safety and replicability. Simultaneously, even the introduction of Ayurveda into the mainstream public health initiatives, such as maternal-child nutrition or school feeding, remains a viable first step. The concerted efforts of Ayurveda, nutrition science, and epidemiology, through the assistance of digital health innovation, will play a significant role in generating evidence that is both scientifically verified and culturally acceptable.

## Conclusions

The review is a new input to the integrative health discussion in that it critically amalgamates Ayurvedic thought with available evidence and community health requirements in the low-resource context. The review combines conceptual background, practice implications, models of community, cost-effectiveness, and policy links into a unifying framework. This comparison of Ayurveda with biomedicine and intersection with the agenda of the WHO on NCDs and the SDGs helps to show how traditional knowledge has informed the development of health interventions that are both universally applicable, sustainable, and culturally applicable. Most importantly, it not only discusses the benefits of Ayurvedic interventions, such as dietary balancing, *Rasayana* therapy, and lifestyle modification, but it also addresses their limitations, i.e., evidence gaps, standardisation, and regulation. It is a synthesis, which tries to create a balance between existing evidence and future research and integration agenda. Thus, the review presents Ayurveda as a context-relevant and complementary, rather than alternative, resource so that it can be an equitable health-promoting resource in at-risk groups of the world population.

## References

[1] Rajeev AM, Malisetty H, Baidya OP, Vamshy J K, Siddhanta S, Dharan BG (2025). Pediatric nutrition and its role in preventing non-communicable diseases: a review. Cureus.

[2] Tumas N, López SR (2024). Double burden of underweight and obesity: insights from new global evidence. Lancet.

[3] Morales ME, Berkowitz SA (2016). The relationship between food insecurity, dietary patterns, and obesity. Curr Nutr Rep.

[4] Arcanjo FP, da Costa Rocha TC, Arcanjo CP, Santos PR (2019). Micronutrient fortification at child-care centers reduces anemia in young children. J Diet Suppl.

[5] Waldman SA, Terzic A (2019). Healthcare evolves from reactive to proactive. Clin Pharmacol Ther.

[6] Lambin P, Zindler J, Vanneste B (2015). Modern clinical research: How rapid learning health care and cohort multiple randomised clinical trials complement traditional evidence based medicine. Acta Oncol.

[7] Wanjarkhedkar P, Sarade G, Purandare B, Kelkar D (2022). A prospective clinical study of an Ayurveda regimen in COVID 19 patients. J Ayurveda Integr Med.

[8] Payyappallimana U, Venkatasubramanian P (2016). Exploring ayurvedic knowledge on food and health for providing innovative solutions to contemporary healthcare. Front Public Health.

[9] Sharma D (2025). Nutrition, lifestyle, and preventive health in Ayurveda: a holistic approach to well-being. Medinity 2025: International Online Conference on Ayurveda and Integrative Medical Sciences.

[10] Gautama PA (2021). RCTs and other clinical trial designs in Ayurveda: a review of challenges and opportunities. J Ayurveda Integr Med.

[11] Goyal M (2018). Lifestyle intervention: a preventive approach for non-communicable diseases. Ayu.

[12] Kathirvel S, Thakur JS (2018). Sustainable development goals and noncommunicable diseases: Roadmap till 2030-a plenary session of the World Noncommunicable Diseases Congress 2017. Int J Noncommunicable Dis.

[13] Wulandari S, Viridula EY, Mayasari W (2025). The impact of community-based yoga on mental health among urban populations: advancing SDG 3 (good health and well-being). J Lifestyle SDG Rev.

[14] (2013). Global Action Plan for the Prevention and Control of Noncommunicable Diseases 2013-2020.

[15] Bharde PS, Shilwant AA (2015). The study of co-relation between rasasarta and rajapravritti. Int Ayurvedic Med J.

[16] Reddy VR, Shitre A (2018). Conceptual study of role of ayurveda in prevention of lifestyle diseases. Int J Res Granthaalayah.

[17] Jain V, Kumar B, Sharma A (2022). A comprehensive yoga programme for weight reduction in children & adolescents with obesity: a randomized controlled trial. Indian J Med Res.

[18] Khan S, Dwivedi A, Jaiswal M (2026). The role of rasayana kalpana in immunomodulation: a scientific and ayurvedic review. J Ayurveda Integr Med Sci.

[19] Shalini T, Ashish G, Umesh S (2020). Malnutrition—a challenge in the 21st century and probable contribution of Ayurveda through Moringa leaves. Int J Res AYUSH Pharm Sci.

[20] Egwumba P, Wang H, Nellums L, Bains M, Chattopadhyay K (2025). Ayurveda for managing noncommunicable diseases in organisation for economic cooperation and development nations: a qualitative systematic review. Health Sci Rep.

[21] Chelak K, Chakole S (2023). The role of social determinants of health in promoting health equality: a narrative review. Cureus.

[22] Jaiswal YS, Williams LL (2017). A glimpse of Ayurveda - the forgotten history and principles of Indian traditional medicine. J Tradit Complement Med.

[23] Rathod T, Rathod K, Tripathi S (2025). Ayurvedic perspective on homeostasis: understanding health through Samayoga. Indian Knowledge Systems of Yoga & Sanskrit for Global Wellbeing.

[24] Hankey A (2005). A test of the systems analysis underlying the scientific theory of Ayurveda's Tridosha. J Altern Complement Med.

[25] Bamane S, Khanolkar G, Madavi K, Shukla DV (2022). A conceptual review of role of Shunthyadi Kwath in Mandagni. World J Pharm Res.

[26] Acharya R (2022). Ayurveda therapeutic and preventive approach: Need for research to create a potential strategy for noncommunicable diseases. J Res Ayurvedic Sci.

[27] Nedungadi P, Salethoor SN, Puthiyedath R, Nair VK, Kessler C, Raman R (2023). Ayurveda research: emerging trends and mapping to sustainable development goals. J Ayurveda Integr Med.

[28] Ansari YA, Rawekar SC, Bandwal PJ (2025). Correlation between awasthapaka and metabolism: an ayurvedic and modern perspective. Int J Res AYUSH Pharm Sci.

[29] Verger EO, Le Port A, Borderon A (2021). Dietary diversity indicators and their associations with dietary adequacy and health outcomes: a systematic scoping review. Adv Nutr.

[30] Rathod P, Jaiswal R (2025). A scientific approach to preventive pregnancy care through Ayurveda classics. Curr Trad Med.

[31] McMillen IC, MacLaughlin SM, Muhlhausler BS, Gentili S, Duffield JL, Morrison JL (2008). Developmental origins of adult health and disease: the role of periconceptional and foetal nutrition. Basic Clin Pharmacol Toxicol.

[32] Raut NR, Mishra BR, Mishra AB (2023). Concept of Hita-Ahita Ahara and its relevance in preservation of good health with special reference to Pathyatam Ahara. J Ayurveda Integr Med Sci.

[33] Sharma K, Garg AK, Chouhan P (2019). Diet and lifestyle modification of winter season (Hemanta and Shishir Ritu): Ayurvedic and modern perspective. World J Pharm Res.

[34] Dhasmana A, Seferidi P, Sharma V, Kohli S, Ghosh-Jerath S (2025). Opportunities for enhancing dietary diversity and environmental sustainability of supplementary nutrition program under the Integrated Child Development Services Scheme - a qualitative study in Srikakulam district, Andhra Pradesh. BMC Public Health.

[35] Khedekar DS, Rathi DR, Rathi DB, Hattikar DH, Patlekar DS (2023). Efficacy of Ayurveda interventions in protein energy malnutrition in children: a systematic review and meta-analysis. Int J Life Sci Pharma Res.

[36] Cömert ED, Gökmen V (2022). Effect of food combinations and their co-digestion on total antioxidant capacity under simulated gastrointestinal conditions. Curr Res Food Sci.

[37] Elegbeleye JA, Fayemi OE, Agbemavor WS (2025). Beyond calories: addressing micronutrient deficiencies in the world's most vulnerable communities-a review. Nutrients.

[38] Agrawal A, Raveendran R, Baranwal S (2022). Ayurvedic preparations for the treatment of iron deficiency anemia: a short review. Indian J Integr Med.

[39] Harinarayan CV, Akhila H (2019). Modern India and the tale of twin nutrient deficiency-calcium and vitamin D-nutrition trend data 50 years-retrospect, introspect, and prospect. Front Endocrinol (Lausanne).

[40] Anju T, Saritha GN, Ramchiary N, Kumar A (2024). Assessing the impact of different cooking methods on nutrients, phytochemicals and antioxidant activity of traditional food plants. Food Chem Adv.

[41] Peñalver R, Martínez-Zamora L, Lorenzo JM, Ros G, Nieto G (2022). Nutritional and antioxidant properties of Moringa oleifera leaves in functional foods. Foods.

[42] Rai VK, Singh V, Rai S (2022). Ayurveda daily regimen practices (Dinacharya): a scientific system model approach suitable as a quaternary prevention strategy for non-communicable diseases. TMR Integr Med.

[43] Jayanna K (2023). Integrative approach to lifestyle management: implications for public health research &amp; practice in the context of SDG-3. J Ayurveda Integr Med.

[44] Balkrishna A, Katiyar P, Upreti J, Chauhan M, Sharma D, Kumar S, Arya V (2025). Investigating Ayurvedic strategies: an in-depth examination of managing diabetes across different types. Curr Diabetes Rev.

[45] Sruthi M, Maindoli G (2024). Rasayana: preventive measures for non-communicable diseases (NCDs). J Ayurveda Integr Med Sci.

[46] Katoch D, Sharma JS, Banerjee S (2017). Government policies and initiatives for development of Ayurveda. J Ethnopharmacol.

[47] Sahu SK, Ghuse RD, Sinha AK, Pandey SK (2021). Role of Achara Rasayana (sublime behaviour) in prevention of non-communicable diseases in India. Int J Ayurveda Pharma Res.

[48] Goyal M (2019). Threats and challenges of emerging viral diseases and scope of Ayurveda in its prevention. Ayu.

[49] Pandi-Perumal SR, Spence DW, Srivastava N (2022). The origin and clinical relevance of yoga nidra. Sleep Vigil.

[50] Priyankisha Priyankisha, Chaudhary V, Soni M (2020). A clinical study to evaluate the efficacy of Amalakavleha as Rasayana in healthy individuals. Ayushdhara.

[51] Semnani-Azad Z, Khan TA, Chiavaroli L (2025). Intermittent fasting strategies and their effects on body weight and other cardiometabolic risk factors: systematic review and network meta-analysis of randomised clinical trials. BMJ.

[52] (2013). WHO Traditional Medicine Strategy: 2014-2023.

[53] Nesari T, Nesari M, Ruknuddin G (2025). India's journey in mainstreaming Ayush in primary health care-from tradition to integration. Front Med (Lausanne).

[54] Alswaidi FM, Abualssayl AA (2025). Ayurveda; safety, effectiveness, and acceptance around the world. F1000Res.

[55] Perera B, Goonaratna C, Ariyawansa H, Senaratna N, Perera J (2025). Efficacy and safety of an Ayurveda herbal formulation in uncomplicated type 2 diabetes mellitus. J Evid Based Integr Med.

